# Endothelial Depletion of Acvrl1 in Mice Leads to Arteriovenous Malformations Associated with Reduced Endoglin Expression

**DOI:** 10.1371/journal.pone.0098646

**Published:** 2014-06-04

**Authors:** Simon Tual-Chalot, Marwa Mahmoud, Kathleen R. Allinson, Rachael E. Redgrave, Zhenhua Zhai, S. Paul Oh, Marcus Fruttiger, Helen M. Arthur

**Affiliations:** 1 Institute of Genetic Medicine, Newcastle University, Newcastle, United Kingdom; 2 Department of Physiology, University of Florida, Gainesville, Florida, United States of America; 3 UCL, Institute of Opthalmology, London, United Kingdom; University of Michigan, United States of America

## Abstract

Rare inherited cardiovascular diseases are frequently caused by mutations in genes that are essential for the formation and/or function of the cardiovasculature. Hereditary Haemorrhagic Telangiectasia is a familial disease of this type. The majority of patients carry mutations in either Endoglin (*ENG*) or *ACVRL1* (also known as *ALK1*) genes, and the disease is characterized by arteriovenous malformations and persistent haemorrhage. *ENG* and *ACVRL1* encode receptors for the TGFβ superfamily of ligands, that are essential for angiogenesis in early development but their roles are not fully understood. Our goal was to examine the role of Acvrl1 in vascular endothelial cells during vascular development and to determine whether loss of endothelial Acvrl1 leads to arteriovenous malformations. Acvrl1 was depleted in endothelial cells either in early postnatal life or in adult mice. Using the neonatal retinal plexus to examine angiogenesis, we observed that loss of endothelial Acvrl1 led to venous enlargement, vascular hyperbranching and arteriovenous malformations. These phenotypes were associated with loss of arterial Jag1 expression, decreased pSmad1/5/8 activity and increased endothelial cell proliferation. We found that Endoglin was markedly down-regulated in Acvrl1-depleted ECs showing endoglin expression to be downstream of Acvrl1 signalling *in vivo*. Endothelial-specific depletion of Acvrl1 in pups also led to pulmonary haemorrhage, but in adult mice resulted in caecal haemorrhage and fatal anaemia. We conclude that during development, endothelial Acvrl1 plays an essential role to regulate endothelial cell proliferation and arterial identity during angiogenesis, whilst in adult life endothelial Acvrl1 is required to maintain vascular integrity.

## Introduction

ACVRL1 (activin receptor like kinase1) is a TGFβ/BMP type I receptor that is required for angiogenesis *in vivo* [1]. It is a membrane protein that is expressed on endothelial cells (ECs), and has a particularly strong affinity for BMP9 and BMP10 ligands [2]. BMP9 (which is present in serum) has been shown to signal through ACVRL1 in ECs and to inhibit angiogenesis in a context specific manner [3], [4]. Furthermore, binding of BMP9 to ACVRL1 (in complex with the BMP type 2 receptor) leads to phosphorylation of SMAD1/5/8 transcription factors which regulate downstream gene expression. Patients who are heterozygous for loss of function mutations in *ACVRL1* develop the vascular disorder Hereditary Haemorrhagic Telangiectasia (HHT) type 2 [5]. A very similar disease (HHT type 1) is caused by mutations in the TGFβ/BMP co-receptor endoglin. Type I and type 2 HHT form the majority of cases of this disease which is characterised by persistent intermittent bleeding from small vascular lesions in the nose and GI tract and by numerous sporadic larger arteriovenous malformations (AVMs) caused by direct connections between arteries and veins that can affect lung, brain and liver [6]. This clinical phenotype points to the importance of ACVRL1 and endoglin in vascular development and in maintaining blood vessel integrity.

When Acvrl1 is depleted from ECs during late foetal development, neonatal mice show systemic enlargement of arteriovenous interconnections, consistent with extensive shunting from arteries to veins [7]. On the other hand, ubiquitous loss of Acvrl1 in adult mice leads to gastrointestinal bleeding that is also a feature of HHT2 patients [7]. Real time imaging following dermal injury revealed *de novo* AVM formation in these mice, and pointed to angiogenesis as a trigger of abnormal vessel remodelling in the absence of Acvrl1 in the adult vasculature [7]. This was recently confirmed in a study that showed VEGF stimulated angiogenesis in the brain led to the development of abnormal vessels resembling AVMs in mice with endothelial specific loss of Acvrl1, but not in controls [8]. Cranial AVMs that develop following loss of Acvrl1 in a zebrafish model were associated with an abnormal increase in endothelial cell proliferation, which occurs partly in response to blood flow [9], [10]. Furthermore, recent work, using adenoviral delivery of the extracellular domain of human ACVRL1 to act as a ligand trap in neonatal mice, showed that removal of BMP9 (and other Acvrl1-binding ligands) in the mouse circulation led to hyperbranching of the retinal plexus, consistent with defects in Notch signalling [11]. However, the effect of losing the endogenous endothelial Acvrl1 receptor in this model has not yet been addressed. We therefore derived a mouse model in which we can trigger endothelial-specific depletion of Acvrl1 in the first week of postnatal life to investigate its role during angiogenesis in vivo. In addition, in the context of HHT, we aimed to compare the vascular phenotype following specific depletion of Acvrl1 from vascular ECs (Acvrl1-iKO^e^) in this study, with the previously reported phenotype resulting from endothelial specific loss of endoglin (Eng-iKO^e^) [12].

## Methods

### Ethics Statement

All animal experiments were performed under UK home office licence with approval from Newcastle University ethical review committee.

### Mice

Floxed *Acvrl1* (*Acvrl1^fl/fl^*) mice, and endothelial-specific tamoxifen inducible Cre line (*Cdh5(PAC)Cre^ERT2^*) were genotyped by genomic PCR as previously described [12]–[15]. Neonatal *Cdh5(PAC)Cre* ^ERT2^;*Acvrl1* ^fl/fl^ mice and *Acvrl1* ^fl/fl^ control littermates were injected subcutaneously with 0.3 mg tamoxifen at postnatal day (P)4 and tissues were harvested at P6, no later than 40 hours post injection. To inactivate Acvrl1 in adult life, *Cdh5(PAC)Cre^ERT2^;Acvrl1^fl/fl^* mice aged 10 to 12 weeks were injected intraperitoneally with 2 mg tamoxifen on two occasions, separated by 1 day; tissues and blood were harvested for analysis 10 days after the first injection. Controls were tamoxifen treated *Acvrl1^fl/fl^* littermates. To reduce variation, only male mice were used in the adult part of the study as there is a gender difference in response to ubiquitous Acvrl1 depletion in adult life [7].

### Retinal immunofluorescence staining and analysis

Mouse eyes were enucleated immediately post-mortem, and retinas prepared and stained as previously described [12], [16]. Essentially retinas were briefly fixed in 4% (w/v) paraformaldehyde in PBS prior to staining. Vascular staining was performed with Alexa488-conjugated B. *Simplicifolia* isolectin B4 (Invitrogen) and immunostaining with primary antibodies to alpha smooth muscle actin (anti-aSMA-Cy3, Sigma), desmin (Millipore), Jag1 (Santa Cruz), CD31 and Endoglin (BD biosciences), EphB4 and Acvrl1 (R&D) [12]. Secondary antibodies conjugated with Alexa594 or Alexa488 (Invitrogen) were used to detect unconjugated primary antibodies. Analysis of branchpoints, filopodia, tip cells and the percentage area covered by isolectin-positive endothelial cells were calculated according to previously reported guidelines [17]. Quantitation of AVM incidence was calculated from fields of view (5300 µm×4460 µm) using images from 24 whole retinas, as previously described [12]. To measure venous diameters, at least 3 veins per retina were measured at 3 positions (200 µm, 250 µm and 300 µm) from the centre of the retina, and 4 retinas were analysed per group. Pericyte coverage of the central vascular capillaries was calculated from 5 fields of view (355 µm×265 µm) per retina using maximum intensity projection confocal images and NIS-Element software (Nikon). Pericyte coverage was calculated as the ratio of area covered by desmin positive muscle cells and normalised to the area covered by CD31-positive endothelial cells. To detect endothelial cell proliferation, pups were injected subcutaneously with thymidine analogues, either 50 µg EdU or 100 µg BrdU per gram body weight two hours prior to harvesting tissues. To visualise EdU positive cells, retinas were stained using Click-iT chemistry according to manufacturer's instructions (Click-iT imaging kit, Invitrogen). To detect BrdU, retinas were treated briefly with 6 mol/L HCl to denature the DNA prior to staining with anti-BrdU-Alexa594 (Invitrogen). A total of 4 fields of view (1420×1060 µm) were analysed in the central region of each retina and the number of proliferating ECs was normalised to isolectin stained vessel area for 6 control and 6 Acvrl1-iKO^e^ retinas. Visualization of ECs with anti-podocalyxin (R&D) to detect the apical endothelial surface and anti-phospho-Smad1/5/8 (NEB) to detect Smad1/5/8 activation was performed following heat-based antigen retrieval on paraffin retinal sections as previously described [18]. Stained retinas were flat mounted in prolong gold mountant including dapi for nuclear staining, (Invitrogen) and examined using a NikonA1R confocal microscope. Quantitation of pSmad1/5/8 staining intensity using NIS-elements software was performed on 56 endothelial nuclei from 3 Acvrl1-iKO^e^ mutant retinas and 65 endothelial nuclei from 3 littermate controls.

### Analysis of Mouse Lung and Caecal Tissue

Lungs and caeca were fixed in 4% paraformaldehyde or formalin, embedded in paraffin, sectioned and stained with haematoxylin and eosin (H&E) and mounted in histomount. Alternatively, paraffin sections were subjected to heat-based antigen retrieval and stained with isolectin B4 and anti-aSMA, then mounted in prolong gold (Invitrogen). Tissues were examined using a Zeiss Axioimager microscope.

### Purification of ECs from tissues

Retinal ECs, or primary ECs from lung tissue, were isolated from wild-type and Acvrl1-iKO^e^ neonates (aged P6). Freshly dissected retinal tissue was finely minced and incubated in 10 mL Dulbecco modified Eagle medium containing 200 U/ml collagenase I (Gibco) for 45 minutes at 37°C. The cells were filtered through a 40 um nylon mesh, recovered by centrifugation (500 g for 5 minutes at 4°C) and resuspended in Buffer 1 (0.1% bovine serum albumin, 2 mM EDTA, pH7.4 in PBS). ECs were incubated with anti-CD31 (BD Pharmingen)-coupled magnetic beads (Invitrogen) for 30 minutes at 4°C and isolated from the cell mixture using a magnetic separator (Dynal). Bead-EC conjugates were washed 5 times with Buffer 1 and centrifuged for 5 minutes at 3400 *g* at 4°C, and the supernatant removed. The purified retinal ECs were then snap frozen and stored at −80°C until required. Total RNA was extracted from purified retinal ECs using the RNAeasy Micro kit (Qiagen). RNA concentration and purity was calculated by measuring the absorbance at 260 and 280 nm using a Nanodrop spectrophotometer (ThermoScientific) and 100 ng of the total RNA was used to prepare template cDNA using RT2 First Strand Kit (Qiagen), according to manufacturers' instructions. Purified ECs from at least 5 Acvrl1-iKOe and 5 control retinas were used to prepare cDNA in four independent experiments using HotStarTaq DNA Polymerase Kit (Qiagen). PCR was used to establish EC purification by comparing Cdh5 and CD31 expression in EC and non-EC fractions after cell sorting. Ve-cadherin (Cdh5) transcripts were detected using forward primer 5′-ACCGTGGGTGTGTGCAAG-3′ and reverse primer 5′-TTTCTTCACGTCGATCATGGTG-3′ and expression of CD31 (Pecam1) was determined using forward primer 5′-AAGCGGTCGTGAATGACAC-3′ and reverse primer 5′-TTTGGCTGCAACTATTAAGGTG-3′. Levels of β-actin were determined using the forward primer 5′-TGAACCCTAAGGCCAACCGTG-3′ and the reverse primer 5′-GCTCATAGCTCTTCTCCAGGG-3′. PCR was performed with 35 cycles of denaturation (30 s at 97°C), annealing (30 s at 58.2°C), and extension (90 s at 72°C), followed by a final extension of 5 min at 72°C for CD31/Pecam1 and β-actin. Cycle conditions for Cdh5 were 35 cycles (97°C, 64°C, 72°C).

Mouse neonatal lung ECs were prepared as previously described [19]. Briefly, lung tissue from pups was cut into small fragments and digested with collagenase/dispase solution (Roche) and dispersed mechanically into single-cell suspension. ECs were purified from cell suspension using anti-CD31 antibody conjugated to Dynabeads using a magnetic particle concentrator and cultured to passage 3 in Endothelial Cell Basal Medium MV2 (PromoCell). Cells were then seeded onto multiwell slides and immunostained for CD31, endoglin and Acvrl1 expression before imaging with a Zeiss Axioimager and digital camera.

### Q-PCR

RNA was prepared from retinal ECs (P6) using the RNAeasy Micro kit (Qiagen) and RNA concentration and purity assessed using a Nanodrop spectrophotometer as above. cDNA was made using RT2 First Strand Kit (Qiagen), according to manufacturers' instructions. Q-PCR was performed using a SYBR Green-based real time PCR custom array (SABioscience) of 85 genes involved in angiogenesis. ECs from at least 5 Acvrl1-iKO^e^ and 5 control retinas were used to prepare cDNA in four replicate experiments for the custom array. For data analysis, the RT2 Profiler PCR Array software was used and statistical analyses performed. This package uses ΔΔCt-based fold change calculations with respect to the average C(t) of 3 housekeeping genes: B2m, Gapdh and Actb and the Student's t-test to calculate two-tail, equal variance p-values.

### Oxygen saturation and blood parameters

Oxygen saturation was measured in adult mice on the day before the first tamoxifen injection by pulse oxymetry (MouseOx; Starr Life Sciences Corp.), and over the subsequent 11 days. The mouse was anesthetised by isoflurane mixed with medical air, and the sensor was placed on a hind limb. Blood chemistry parameters were obtained using blood harvested by cardiac puncture from anesthetised adult mice (10 days after the first tamoxifen injection) and analysed on a CC8+ cartridge (Abaxis) using an i-STAT portable reader (Abbott Laboratories, Princeton, NJ).

### Statistical Analysis

Data were analysed by two-way analysis of variance, and paired Student's t-test using Graphpad Prism (version 5, Graphpad Software Inc., La Jolla, CA, USA). Data are expressed as mean ±SEM, and and p<0.05 was considered to be statistically significant.

## Results

### Endothelial specific depletion of Acvrl1 in neonates leads to rapid mortality associated with pulmonary haemorrhage

**Figure 1:**
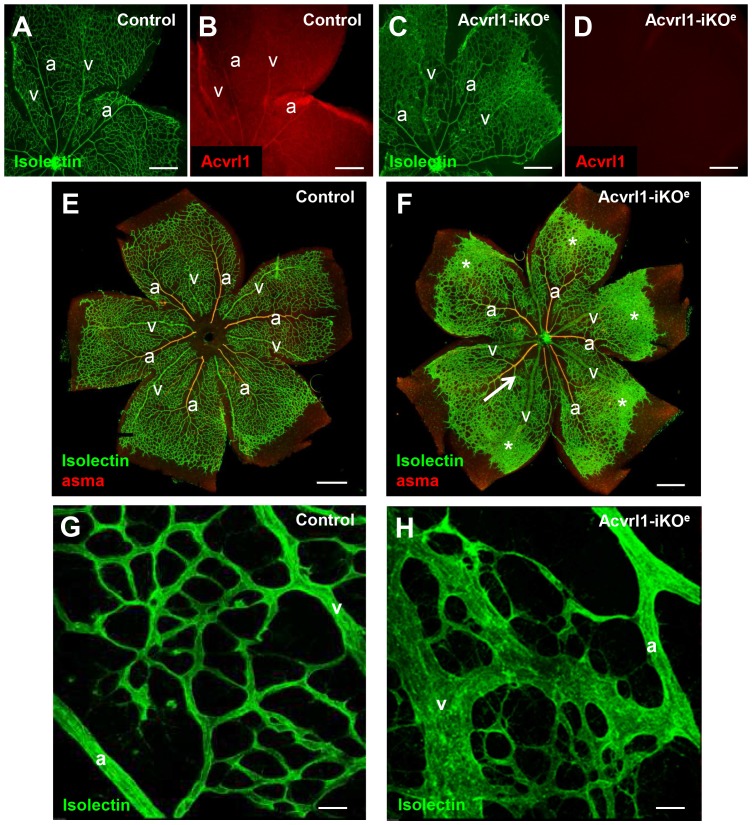
Loss of endothelial Acvrl1 expression leads to abnormalities in the neonatal retinal vascular plexus. Acvrl1 expression in control retinas at P6 is seen in veins, arteries and capillaries (A,B) and is efficiently knocked down 40 hours after tamoxifen injection (C,D). Loss of Acvrl1 protein in endothelial cells in the Acvrl1-iKO^e^ mouse leads to AVMs (F, arrow), enlarged veins (compare veins in F and E as well as H and G) and hyperbranching (F, asterisks). Arteries are muscularised in both Acvrl1-iKO^e^ and control retinas, as indicated by staining for alpha smooth muscle actin (aSMA) positive smooth muscle cells (E,F). An AVM at higher magnification (H) contrasts with the normal capillary network seen in control retinas (G). Abbreviations a, artery; v, vein. Scale bar = 400 µm A–D; 500 µm E and F; 25 µm G and H.

Acvrl1 is expressed in the veins, arteries and capillaries of the neonatal retina at postnatal day (P) 6 (Figure 1A,B). Tamoxifen injection of *Cdh5(PAC)Cre^ERT2^;Acvrl1^fl/fl^* neonatal mice led to efficient loss of Acvrl1 protein expression in the neonatal retina to generate Acvrl1-iKO^e^ mice (Figure 1C,D). By 48 hours post injection, Acvrl1-iKO^e^ showed severe respiratory distress leading to rapid mortality. The timing was therefore optimised to examine neonates at 40 hours post tamoxifen injection, which was after Acvrl1 protein depletion, but before the severe adverse symptoms began. Examination of Acvrl1-iKO^e^ neonatal lungs revealed haemorrhage from the distal small capillaries, which was absent in controls, but we observed no detectable loss of vascular smooth muscle cells (vSMCs) from the pulmonary blood vessels (Figure S1).

### Endothelial specific depletion of Acvrl1 in neonates leads to retinal arteriovenous malformations and hyperbranching

**Figure 2:**
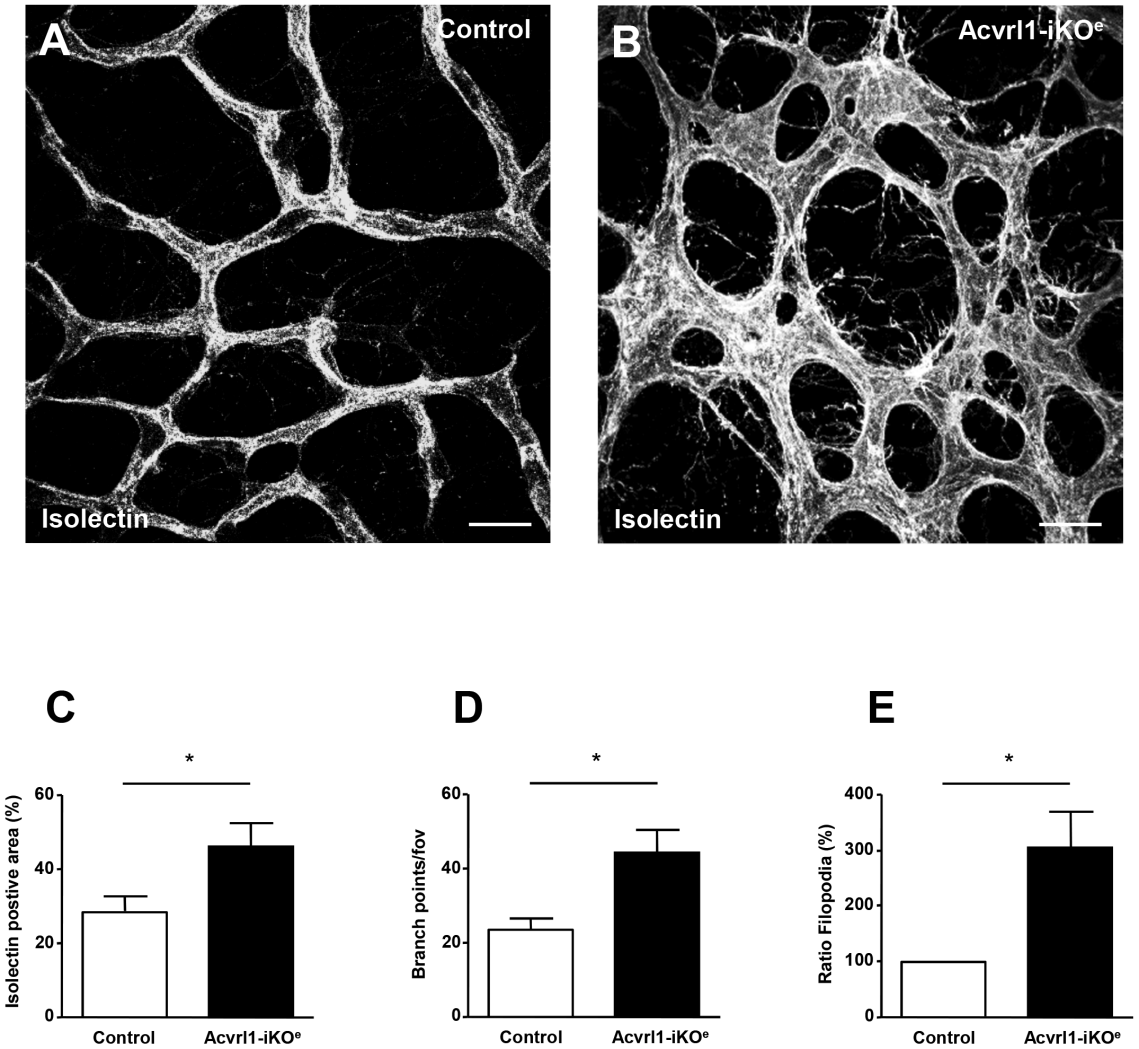
Hypervascularity of the Acvrl1-iKO^e^ retinal plexus. Neonatal Acvrl1-iKO^e^ retinas show increased vascular branching compared with controls (A,B). Vessel density (C), vessel branch points (D), and density of filopodia (E) are all significantly increased in Acvrl1-iKO^e^ retinas (P6) compared with controls. *p<0.05. Scale bar = 25 µm.

**Figure 3:**
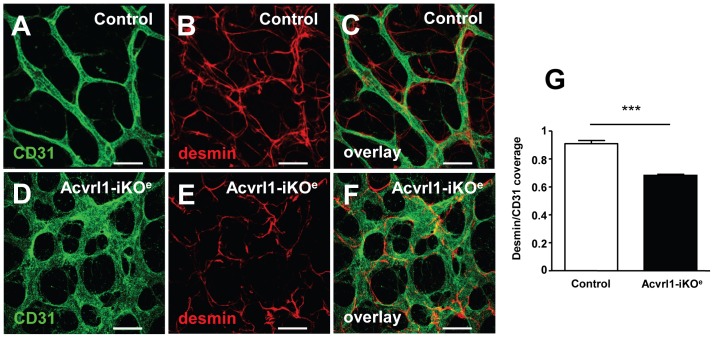
Reduced pericyte coverage of capillaries in neonatal Acvrl1-iKO^e^ retinas. Desmin staining revealed that pericyte coverage of capillaries in Acvrl1-iKO^e^ retinas (D–F) was reduced compared with controls (A–C). Quantitation of the ratio of desmin to CD31 staining confirmed that the increased endothelial cell density in Acvrl1-iKO^e^ retinas was not accompanied by an equivalent increase in pericyte density (G). *p<0.05; ***p<0.001. Scale bar = 20 µm A,B; 25 µm D-I.

We chose to investigate the effect of endothelial depletion of Acvrl1 in the retinal vasculature using a similar approach to one we had used previously [12]. This approach has a number of advantages. First, the neonatal retinal plexus undergoes a well characterised process of vascularisation in the first week of life allowing identification of any aberrant angiogenesis events in the absence of endothelial Acvrl1 [20]. Second, individual endothelial cells can be visualised within a simple two dimensional vascular permitting analysis of key parameters such as cell proliferation, smooth muscle coverage, arterial and venous identity as well as cell-specific signalling in vivo. Using this approach we identified numerous vessel abnormalities in the Acvrl1-iKO^e^ mutants compared with controls, including AVMs, enlarged veins and hyperbranching of the capillary plexus (Figure 1E–H). Approximately 60% of Acvrl1-iKO^e^ neonatal retinas showed AVMs (14/24 retinas); and when present there were usually multiple AVMs (average number per retina = 2.7). Even more frequently, enlarged veins were observed in 70% of the Acvrl1-iKO^e^ retinas (Figure 1F & H); the mean vein width was 23.99+/− 1.4 µm in control retinas and 42.62+/−2.3 µm in Acvrl1-iKO^e^ retinas (p<0.001). Increased vascular branching (100% incidence) was associated with higher vascular density (Figure 2B) and greater numbers of filopodia, consistent with the presence of increased numbers of tip cells in the central region of the plexus, an area where the tip cell phenotype is normally suppressed (Figure 2). There was no significant difference between Acvrl1-iKO^e^ and control retinas in progression of the plexus towards the edge of the retina (Figure S2) suggesting there was no defect in migration. Pericyte coverage of the arteries and veins was normal in Acvrl1-iKO^e^ retinas, but capillaries showed decreased pericyte coverage in Acvrl1-iKO^e^ mutants compared with controls (Figure 3).

**Figure 4:**
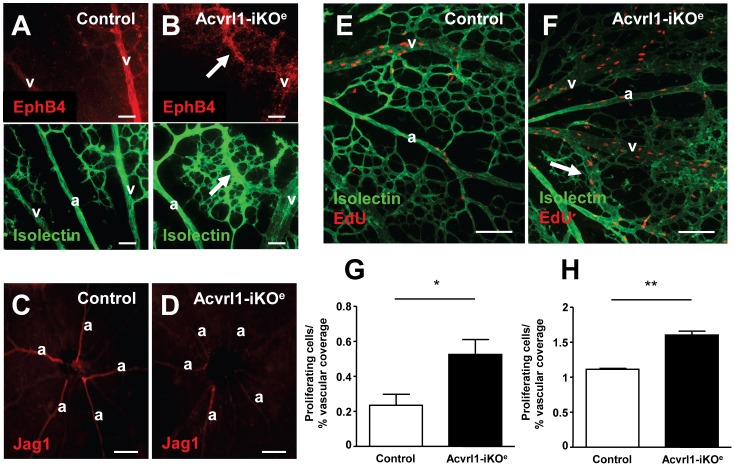
Loss of arterial identity and increased EC proliferation in neonatal Acvrl1-iKO^e^ retinas. Retinal veins show retention of venous marker EphB4 similar to control (A,B) and AVMs are also EphB4 positive (arrow, B). Jag1 expression in arteries of Acvrl1-iKO^e^ retinas is reduced compared with controls (C,D). EdU labelling (E,F) reveals a significant increase in EC proliferation in mid plexus capillaries (G) and veins (H) of Acvrl1-iKO^e^ retinas compared with controls. Increased EC proliferation is also associated with AVMs in Acvrl1-iKO^e^ mutants (arrow, F). *p<0.05; **p<0.01. Scale bar = 50 µm A and B; 200 µm C and D; 100 µm E and F.

As AVMs can be caused by loss of arterial and venous identity, we next investigated whether arteries and veins of the Acvrl1-iKO^e^ retinal plexus retained expression of key venous and arterial molecular markers. We found that expression of the venous marker, EphB4, was retained in the veins of Acvrl1-iKO^e^ retinas (Figure 4A,B), but there was markedly reduced jagged 1 (Jag1) expression in the mutant arteries compared with controls, suggesting loss of arterial identity (Figure 4C,D). Interestingly, AVMs in the Acvrl1 deficient retinas also expressed the venous marker EphB4, consistent with AVMs possessing a venous identity (Figure 4B). Using thymidine analogues to monitor proliferating cells we observed there was increased EC proliferation in AVMs, as well as in the enlarged veins, in comparison with littermate controls, pointing to an abnormal increase in EC proliferation underlying the enlargement of both veins and AVMs in Acvrl1-iKO^e^ retinas (Figure 4E–H).

### Endothelial specific depletion of Acvrl1 in neonates leads to reduced phospho-activation of Smad1/5/8 and changes in downstream gene expression

**Figure 5:**
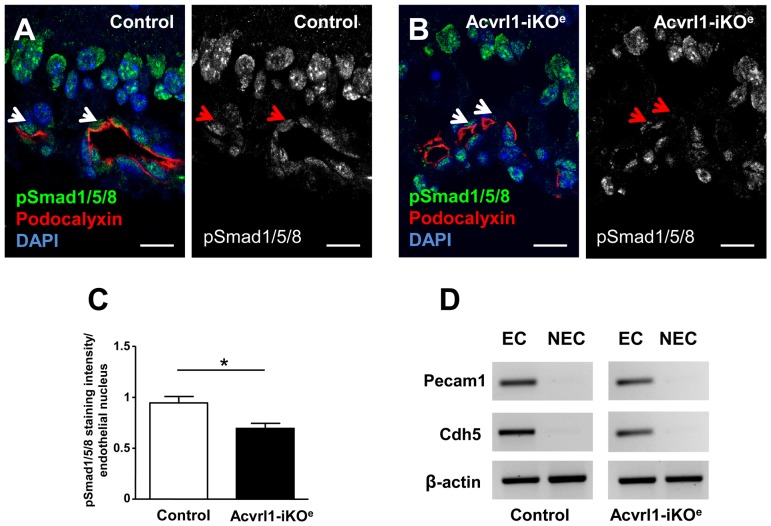
Reduced pSmad1/5/8 activity and loss of endoglin expression in endothelial cells of neonatal Acvrl1-iKO^e^ retinas. Retinal sections stained for pSmad1/5/8 (green) reveal Smad1/5/8 activation in vascular cells and neural cells in control retinas (A). Confocal analysis of podocalyxin staining (red) was used to identify the apical surface of endothelial cells in retinal blood vessels. Reduced pSmad1/5/8 staining can be seen in endothelial cells in Acvrl1-iKO^e^ retinas (B), and was quantified using confocal software. Statistical analysis of pSmad1/5/8 staining intensity shows a significant reduction in endothelial cells of Acvrl1-iKO^e^ mutants compared with controls (C). Expression of pan-endothelial markers by rtPCR was used to confirm retinal endothelial cell (EC) purification by antibody conjugated magnetic beads. Pecam1 and Cdh5 were detected in the cDNA prepared from EC fractions compared to the non-EC (N-EC) fractions prepared from Acvrl1-iKOe and control retinas. Expression of β-actin was used as a positive control.

**Figure 6:**
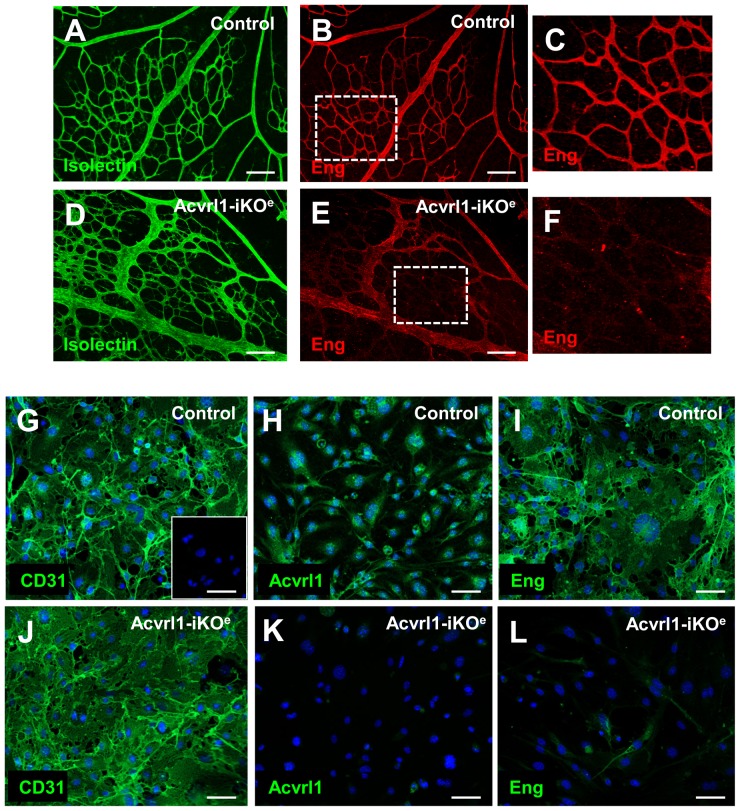
Endothelial cells from Acvrl1-iKO^e^ mice show loss of endoglin expression. Endoglin expression was reduced in Acvrl1-iKOe retinas (E) compared with controls (B) and this was particularly marked in the capillaries. Representative capillary regions indicated in B and E are shown in digital zoom in C and F, respectively. *p<0.05. Scale bar = 20 µm A–B; 100 µm D,E,F,H. Purified lung endothelial cells from control (Acvrl1^fl/fl^) and Acvrl1-iKO^e^ neonatal mice were immunostained for the pan endothelial marker CD31 to confirm endothelial cell purity (G,J). Cells from the Acvrl1-iKO^e^ mice showed not only loss of Acvrl1 protein (K), but also reduced endoglin expression (L) compared with controls (I). Dapi was used to stain cell nuclei and inset in G shows no primary antibody control. Scale bar = 50 µm.

As Acvrl1 normally responds to circulating ligands by phosphorylating Smad1/5/8, we used antibodies to detect the phosphorylated form of Smad1/5/8 in Acvrl1-KO^e^ retinas and found that pSmad1/5/8 activity was significantly reduced in Acvrl1-KO^e^ ECs compared with controls (Figure 5A–C). We therefore aimed to test whether there was a reduction in Id1, Id2 or Id3 transcripts, which lie downstream of this pathway. To do this we compared the relative transcript levels in purified retinal ECs of Acvrl1-iKO^e^ mutants and controls using a custom SABiosciences qPCR array. First, we established a method to reproducibly purify neonatal retinal endothelial cells and confirmed endothelial cell enrichment using rtPCR for pan endothelial markers Cdh5 and CD31 (Figure 5D). Retinal ECs were then used to evaluate changes in expression of a range of genes (Table S1) that had either been reported to be dysregulated following loss of Acvrl1 [10], [21] or were downstream of BMP9 signalling [22]–[24] or were associated with a related phenotype. Of the 85 candidate genes tested, 14 were found to be significantly down-regulated by over 50%, but no genes were up regulated (Table 1). Acvrl1 transcripts were reduced by 5.7 fold, consistent with efficient gene knockdown, and Id3 (but not Id1 or Id2) was downregulated 3.6-fold, in agreement with the observed reduction in pSmad1/5/8 activity. Expression of Notch1 and Flt1 were reduced in line with the altered tip cell phenotype [25], [26], and the vasoregulators eNOS and Ptgs2 were also downregulated. Reduced expression of three key transcription factors, FoxC1, FoxC2 and Sox17, each with known roles in the regulation of angiogenesis [27], [28], was also observed. However, at 33-fold reduction, endoglin was the most dramatically downregulated transcript in the array. Endoglin depletion in Acvrl1-iKO^e^ neonatal retinas was confirmed at the protein level and was particularly evident in capillary ECs of the retina (Figure 6A–F). This effect was also seen in other tissues: analysis of neonatal pulmonary ECs from Acvrl1-iKO^e^ and control mice confirmed reduced endoglin expression following depletion of Acvrl1 (Figure 6 G–L).

**Table 1:** Changes in transcript levels in ECs following knockdown of Acvrl1 expression.

Gene	Fold Down-regulation	P value	Potential Role	Reference
*Acvrl1*	5.7	0.0029	Target gene	
*Eng*	33.4	0.0029	Endoglin promotes Acvrl1 signalling	[41]
*Id3*	3.59	0.038	Transcription factor downstream of pSmad1/5 signalling	[41]
*FoxC1*	4.61	0.038	Regulate angiogenesis and lymphangiogenesis	[27]
*FoxC2*	2.97	0.011		
*Fzd4*	3.04	0.038	Involved in neural angiogenesis	[28]
*Sox17*	2.29	0.011		
*Lrp5*	1.8	0.029		
*Nos3*	2.42	0.033	Regulate vascular tone, downregulated in endoglin mutants.	[46], [47]
*Ptgs2*	1.9	0.004		
*Sele*	1.98	0.011	Regulated by BMP9 and involved in leucocyte interactions with ECs.	[24]
*Flt1*	1.57	0.032	Production of sFlt1 a ‘ligand sink’ for VEGFA, required for efficient lateral Notch signalling	[53]
*VegfC*	3.82	0.0075	Regulates lymphangiogenesis.	[54]
*Notch1*	3.95	0.036	Regulates tip cell-stalk cell phenotype in angiogenesis	[25]

Quantitative PCR using a custom array of 85 genes involved in angiogenesis revealed changes in transcript levels of the 14 listed genes.

### Endothelial specific depletion of Acvrl1 in adult life leads to caecal haemorrhage

To investigate the role of Acvrl1 in ECs in adult life, we also examined the vasculature of adult retinas following EC-specific depletion of Acvrl1 in mice aged between 10 and 12 weeks. Loss of Acvrl1 in adult retinal ECs had no detectable effect on vessel organisation or vessel branching, consistent with a requirement for angiogenesis to generate AVMs (Figure S3). However, approximately 10 days after Acvrl1 depletion was initiated, these mice developed severe GI bleeding, evident as black faeces (Figure S4) in a similar way to previously reported for the ubiquitous Acvrl1 knockout mouse [7]. Post mortem examination at day 11 revealed the source of bleeding was located to fragile microvessels in the villi of the caecum (Figure S4). To investigate the effect of blood loss on oxygen transport to the peripheral vasculature, mice were monitored pre- and post- tamoxifen treatment for oxygen saturation which was found to be significantly reduced at day 11 compared with controls (Figure S4). Cardiac (left ventricle) blood parameters were measured at day11 and the mean haematocrit level was 35.6% of packed cell volume in control mice but was significantly reduced to 13.2% (p<0.01) in Acvrl1-iKO^e^ mice. Similarly, the mean haemoglobin levels were 12 g/dL in controls, but only 4.5 g/dL in the Acvrl1-iKO^e^ (p<0.01), suggesting that haemorrhage was the major cause of low peripheral oxygen saturation (Table S2). However, blood gas analysis of cardiac left ventricular blood from Acvrl1-iKO^e^ mutants showed that the oxygen saturation was unchanged (Table S2), suggesting that pulmonary AVMs were either absent or asymptomatic *in vivo*.

## Discussion

We have shown that loss of Acvrl1 from ECs in neonates leads to a significant increase in vessel branching and increased numbers of tip cells in the angiogenic neonatal retinal plexus, similar to the retinal phenotype observed when an ACVRL1 ligand trap was used to reduce Acvrl1 signalling [11]. This hyperbranching phenotype was also observed following depletion of BMP10 in retinas of the BMP9 null mouse [29], pointing to BMP9 and BMP10 as the activating ligands for Acvrl1 signalling *in vivo*. Furthermore, increased numbers of tip cells and hyperbranching resulted from endothelial-specific depletion of Smad1 and Smad5 in developing embryos [30]. In agreement with this, we observed that neonatal Acvrl1-iKO^e^ retinas also showed reduced endothelial pSmad1/5/8 levels. Taken together, these findings are mutually supportive and consistent with BMP9/10 signalling through Acvrl1 to activate Smad1/5/8 to synergise with Notch signalling. This acts to minimise tip cell formation in the stalk and phalanx ECs during angiogenesis in vivo [11], [30], [31]. In addition, our data suggests that Acvrl1 activity would also affect Notch signalling by regulating Notch1 and Jag1 expression.

The interaction of Acvrl1 and Notch signalling defects during the development of AVMs is less clear. Firstly, both loss and gain of Notch signalling can lead to AVM formation. For example, Notch mutant embryos develop AVMs, thought to be due to fusions of veins and arteries following loss of arterial and venous identity [32]. On the other hand, upregulation of Notch4 or Notch1 signalling also leads to AVM formation [33], [34]. However, AVMs that develop following increased Notch4 signalling are reversible once excessive Notch signalling is normalised [34], but it remains to be determined to what extent the reversible AVMs are a feature of arteriovenous fusion, dysregulated vascular smooth muscle tone or excess endothelial cell proliferation. In HHT, it is not clear whether AVMs form as a result of lost arteriovenous identity. From analysis of the mouse models we can say that AVMs are a feature of Eng-iKO^e^ retinas where arteriovenous identity was maintained. In contrast, the Acvrl1-iKO^e^ mutant retinas show a striking reduction in arterial Jag1 expression. This finding is consistent with a previous report showing that Jag1 expression is regulated by BMP9 [22] and agrees with the loss of arterial identity that was first reported in *Acvrl1* null mouse embryos [35]. In contrast, venous identity is preserved, as judged by retention of EphB4 expression in the Acvrl1-iKO^e^ retinal veins. Furthermore, AVMs in the Acvrl1-iKO^e^ neonatal retinas also expressed EphB4, consistent with a venous identity for these arteriovenous connections. In contrast to a previous report [7] we observed that loss of endothelial Acvrl1 did not lead to detectable loss of vascular smooth muscle cells. This difference is likely to be because we are only examining events that occur within 40 hours of tamoxifen treatment, whereas the previous study involved Acvrl1 depletion from approximately E17 to P3 providing more opportunity for pulmonary vessel remodelling to occur [7]. However, we did confirm the microvessel haemorrhage seen in the postnatal lungs following loss of Acvrl1 [7], demonstrating the endothelial cause of this fragility defect.

The majority of HHT patients carry mutations in either endoglin (HHT type 1) or *ACVRL1* (HHT type 2) genes [5], [36]. Retinal vascular malformations have been reported in HHT patients although they are not considered a major clinical problem [37], [38]. Both HHT type 1 and HHT type 2 patient groups have a similar clinical presentation, suggesting that endoglin and ACVRL1 perform a similar function. Endoglin is a co-receptor for the TGFβ superfamily, has a high binding affinity for BMP9 [39], [40], and promotes signalling through ACVRL1 [41]. Examination of the neonatal retinal plexus following loss of endothelial endoglin (Eng-iKO^e^) [12] or Acvrl1 (this study) has revealed interesting similarities and differences. In both cases, increased endothelial cell proliferation contributed to the AVMs, which also showed venous identity. The AVMs developed in a similar way but there was no loss of arterial identity in the Eng-iKO^e^, suggesting that loss of arterial or venous identity is not a prerequisite for AVM formation in HHT. Furthermore, Eng-iKO^e^ retinas differed from Acvrl1-iKO^e^ retinas in that they did not exhibit hyperbranching or show altered pSmad1/5/8 levels, suggesting that some BMP9/10 signalling was preserved in the absence of endothelial endoglin, and consistent with the role of endoglin as a facilitator of ligand binding, rather than being essential for signalling. Furthermore, loss of endothelial Acvrl1 in our study leads to a rapid lethality, which appeared to be due to microhaemorrhage of the lung, whereas Eng-iKO^e^ neonates are viable.

**Figure 7:**
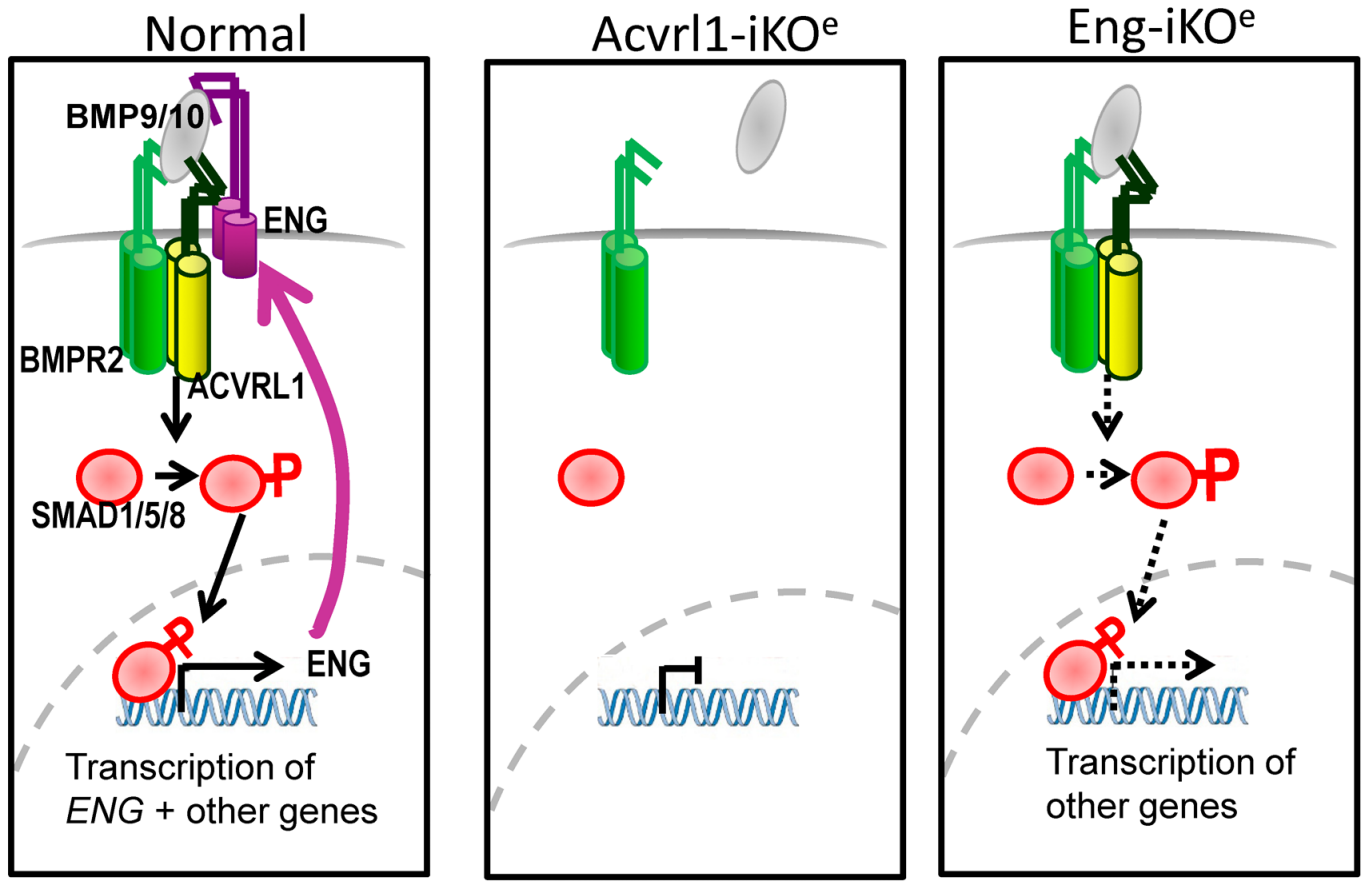
Diagrammatic Summary of Normal and Disrupted Signalling following Acvrl1 Depletion in Endothelial Cells. In normal endothelial cells endoglin promotes BMP9/10 signalling through the ACVRL1/BMPR2 receptor complex (as well as the ACVRL1/TGFBR2 complex, not shown). ACVRL1 phosphorylates SMAD1/5/8 which is then able to move to the nucleus (in combination with SMAD4) to regulate downstream expression of many genes. BMP9 signalling leads to increased endoglin expression [22]–[24] which in turn promotes ACVRL1 signalling in a positive feedback loop. In the absence of ACVRL1, Smad1/5/8 signalling is reduced and endoglin is no longer expressed. On the other hand, when endoglin is depleted from endothelial cells, residual signalling through Acvrl1 is able to proceed, but at a lower efficiency.

There was a striking reduction of endoglin expression in Acvrl1-iKO^e^ mice so that reduced endoglin expression is a common feature of both Eng-iKO^e^ and Acvrl1-iKO^e^ genotypes. This finding agrees with the reduced endoglin expression previously reported in blood outgrowth ECs and in activated monocytes from HHT type 2 patients [21], [42], although endoglin expression did not appear to be reduced in HUVECs or activated monocytes from HHT2 patients in an earlier study [43]. As endoglin expression is downstream of BMP9/10 signalling, [22]–[24] then reduced Acvrl1 activity would explain the decrease in endoglin expression. This finding is compatible with a positive feedback loop: endoglin promotes Acvrl1 signalling [41] and is also expressed downstream of Acvrl1 signalling. We observed no reciprocal downregulation of Acvrl1 expression in Eng-iKO^e^ mutants (data not shown). Together these findings suggest that BMP9/10 signalling is not sufficiently disturbed to reduce pSmad1/5/8 in Eng-iKO^e^ mice and that Acvrl1 is more critical for BMP9/10 signalling *in vivo*. Figure 7 shows a diagrammatic summary of the disrupted signalling pathway in Acvrl1-iKO^e^ and Eng-iKO^e^ mice. Intriguingly, it is also possible that the features common to both Eng-iKO^e^ and Acvrl1-iKO^e^ phenotypes such as AVM formation (summarised in Table 2) may be primarily due to loss of endoglin. This hypothesis could be tested in future studies by restoring endoglin expression to Acvrl1-iKO^e^ mice using a transgenic or related approach.

**Table 2:** Summary of key differences in the neonatal retinal vascular plexus when either Acvrl1 or Eng is depleted from endothelial cells.

Phenotype	Acvrl1-iKO^e^	*Eng-iKO^e^
AVMs	Present	Present
Vessel Branching	Increased	Increased only at periphery
Angiogenesis Delay	No	Yes
Venous Identity	Preserved, but veins enlarged	Preserved, but veins enlarged
Arterial Identity	Reduced	Preserved
Endothelial Cell Proliferation	Increased	Increased
Smad1/5 Phosphorylation in Endothelial Cells	Reduced	No change detected

NB All relative terms (eg ‘increased’ and ‘reduced’) are used with respect to these features in normal (control) retina. *See reference [12] for further details of the Eng-iKO^e^ phenotype.

In addition to Acvrl1 and endoglin, 83 candidate genes were examined for changes in transcript levels in Acvrl1-iKO^e^ retinal ECs (Table S1) and 12 of these genes were downregulated. Reduced Notch1 expression may contribute to decreased Notch signalling in these retinas [25], but we were unable to detect a significant change in transcript levels of the Notch immediate downstream effector genes, Hes1 and Hey1 *in vivo*. However, we did observe reduced VegfC levels that may also contribute to the lymphangiogenesis defects seen in mice treated with an Acvrl1 ligand trap [44]. Further studies comparing vascular-specific and lymphatic-specific Acvrl1 knockouts are required in order to determine their relative contributions to VegfC transcript levels. Furthermore, expression of FoxC1 and FoxC2, which encode transcription factors involved in regulation of angiogenesis upstream of Notch, are also reduced in Acvrl1-iKO^e^ retinas and may contribute to Notch signalling defects. Moreover, FoxC2 is regulated by BMP9 and plays a role in lymphatic development [45]. It is also relevant here that compound knockouts for FoxC1 and FoxC2 transcription factors result in mouse embryos with AVMs resulting from a fusion of veins and arteries similar to those seen in Acvrl1 null embryos [27], [35]. Levels of eNos and Ptgs2 (also known as Cox2) transcripts were downregulated 2.4 and 1.9 fold, respectively, in Acvrl1-iKO^e^ retinal ECs. These genes are both involved in vasomotor control and have been reported as reduced in Endoglin deficient mice [46], [47]. We also observed reduced pericyte coverage of the Acvrl1-iKO^e^ retinal capillaries, similar to a previous report describing local Acvrl depletion in adult cerebral vessels exposed to angiogenic stimuli [48]. However, we detected no significant reduction in Pdgfb expression in our custom array analysis (not shown), so the pericyte defects in our model may therefore relate to the increased proliferative status of the Acvrl1-iKO^e^ ECs rather than defects in Pdgf signalling.

In contrast to neonatal Acvrl1-iKO^e^ mice, no AVMs were detected in retinas of adult Acvrl1-iKO^e^ mice, consistent with our hypothesis of angiogenesis-dependent AVM formation. However, bleeding from the caecum, a pouch like structure that lies between the ileum and colon, was sufficiently severe to cause a fatal anaemia. Even though the caecum is much smaller in human than mouse, caecal AVMs have been reported in HHT [49] although GI bleeding in HHT patients is more frequently associated with the stomach and upper duodenum [50]. It is not clear why different vascular beds are susceptible to bleeding in HHT, but loss of endothelial Acvrl1 in the mouse leads to increased vessel fragility even in fully formed vessels of the adult caecum, whereas AVM formation in the retina occurs only in the context of developmental angiogenesis and vessel remodelling.

In conclusion, this study reveals the importance of endothelial Acvrl1 signalling in regulating vascular branching and endothelial cell proliferation during angiogenesis in vivo. We also confirm the angiogenesis dependence of AVM formation. In adult HHT patients, inflammation is likely to be the most frequent trigger of angiogenic responses, and therefore anti-inflammatory drugs may play a protective role in controlling lesion development. Our findings also impact on the wider role of ACVRL1 in regulating cardiovascular pathophysiology [51] and the development of anti-ACVRL1 therapies[52] for treating cancer patients.

## Supporting Information

Figure S1 **Lung defects in neonatal Acvrl1-iKO^e^ mice.** H&E stained lung sections show extensive haemorrhage in the lung capillaries of Acvrl1-iKO^e^ pups at P6 (B) compared with age matched controls (A). However there was no detectable loss of supporting vascular smooth muscle cells in the pulmonary blood vessels (B,C). Lung vasculature was revealed using isolectin staining (green) and smooth muscle cells were detected using anti-alpha smooth muscle actin (aSMA, red). Abbreviations: a, artery; br, bronchiole; v, vein. Scale bar = 100 um.
(TIF)

Figure S2 **No reduction in progression of the retinal vascular plexus in neonatal Acvrl1-iKO^e^ retinas.** The relative distance covered by the vascular plexus was calculated as the ratio of the vascular radius, indicated by the blue arrow, and the retinal radius, indicated by the red arrow in 3 separate regions for each retina. The size of each retina was calculated as the mean of the retinal radii and mean values are shown +/− standard error. Scale bar = 500 um.
(TIF)

Figure S3 **Normal vasculature of adult Acvrl1-iKO^e^ retinas.** No vascular abnormalities were detected in vessels from Acvrl1-iKO^e^ (D–E) adult retinas compared with control retinas (A–C). Acvrl1 was depleted in endothelial cells during adult life and retinas were stained for vascular smooth muscle cells using anti-aSMA (red) and for ECs with isolectin (green). Scale bar = 500 um.
(TIF)

Figure S4 **Adult Acvrl1-iKO^e^ mice develop GI bleeding from the caecum.** Tamoxifen was given to adult mice (on day 1 and day 3 as indicated by black arrows) to generate Acvrl1-iKO^e^ mice. Peripheral oxygen saturation was significantly reduced in Acvrl1-iKO^e^ adult mice at day 11* p<0.05 (A). GI bleeding was first observed by black faeces (inset in D), compare with normal faeces (inset in C), approximately 9 days after the first tamoxifen injection. Analysis of the GI tract showed bleeding was localised to the caecum, which appeared intensely dark coloured (D) compared with the caecum from control mice (C). Furthermore, the contents of the small intestine proximal to the caecum were normal in colour (asterisk in D), whilst the content of the large intestine immediately distal to the caecum was much darker indicating a significant blood content (arrow in D). H&E stained caecal sections revealed bleeding from fragile vessels in the caecal villi of adult Acvrl1-iKO^e^ mice (arrows, F).
(TIF)

Table S1 **Genes used in custom array qPCR analysis to evaluate changes in transcription following loss of Acvrl1.** (* indicates housekeeping genes used for data normalisation).
(DOCX)

Table S2 **Blood Gas Analysis of Acvrl1-iKO^e^ Adult Mice.** Blood from adult male Acvrl1-iKO^e^ and control mice (aged 12 weeks) was taken by cardiac puncture under terminal anaesthesia and analysed using a CG8+ cartridge with an Istat portable reader. Blood values are expressed as mean ±SEM.
(DOCX)
