# Supplementary figures and images for: Endothelial Depletion of Acvrl1 in Mice Leads to Arteriovenous Malformations Associated with Reduced Endoglin Expression
Source: PLoS One. 2014 Jun 4;9(6):e98646. doi: 10.1371/journal.pone.0098646 (PMC4045906; doi:10.1371/journal.pone.0098646)

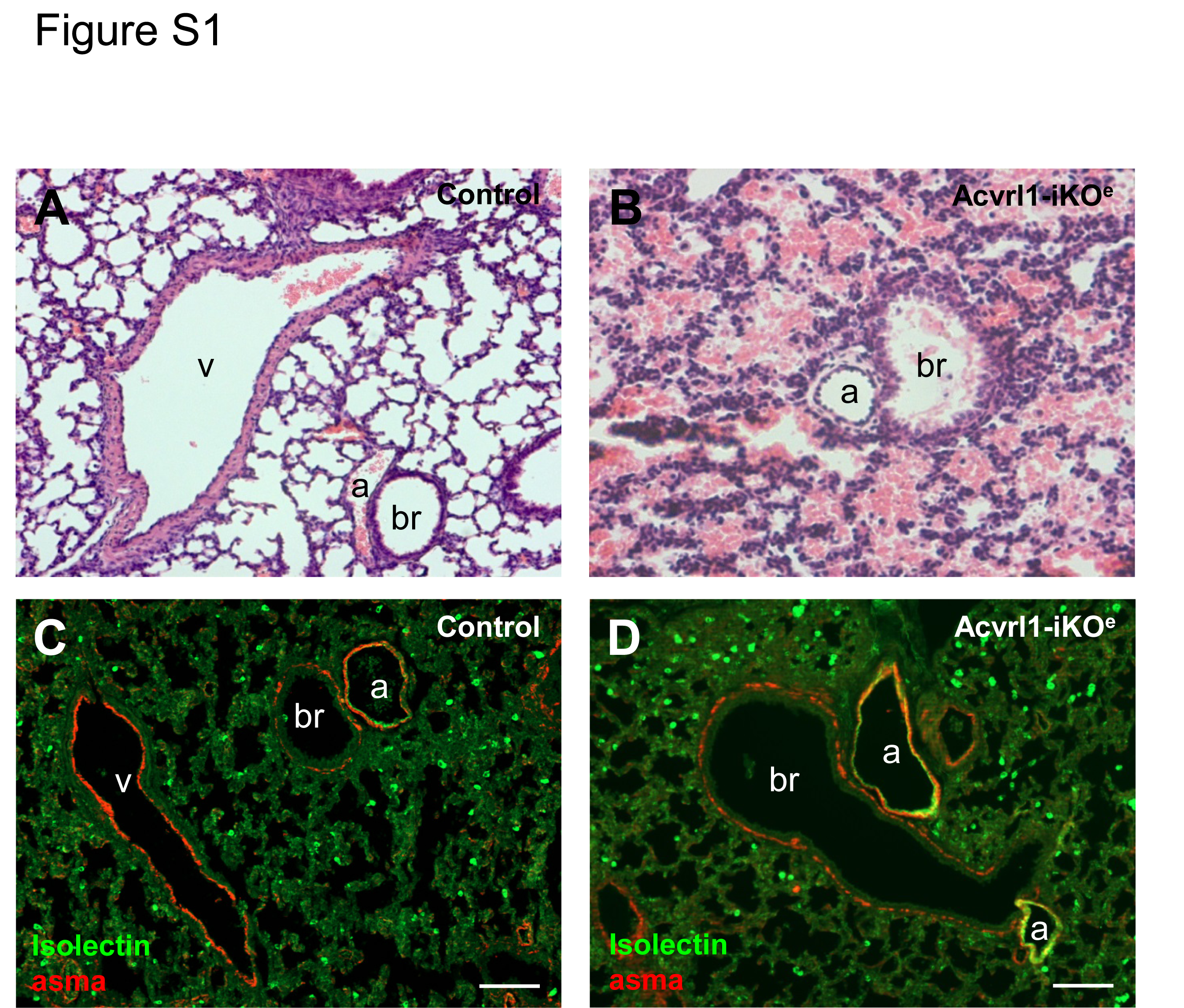

Supplement: Figure S1 — Lung defects in neonatal Acvrl1-iKOe mice. H&E stained lung sections show extensive haemorrhage in the lung capillaries of Acvrl1-iKOe pups at P6 (B) compared with age matched controls (A). However there was no detectable loss of supporting vascular smooth muscle cells in the pulmonary blood vessels (B,C). Lung vasculature was revealed using isolectin staining (green) and smooth muscle cells were detected using anti-alpha smooth muscle actin (aSMA, red). Abbreviations: a, artery; br, bronchiole; v, vein. Scale bar = 100 um. (TIF) [file pone.0098646.s001.tif]

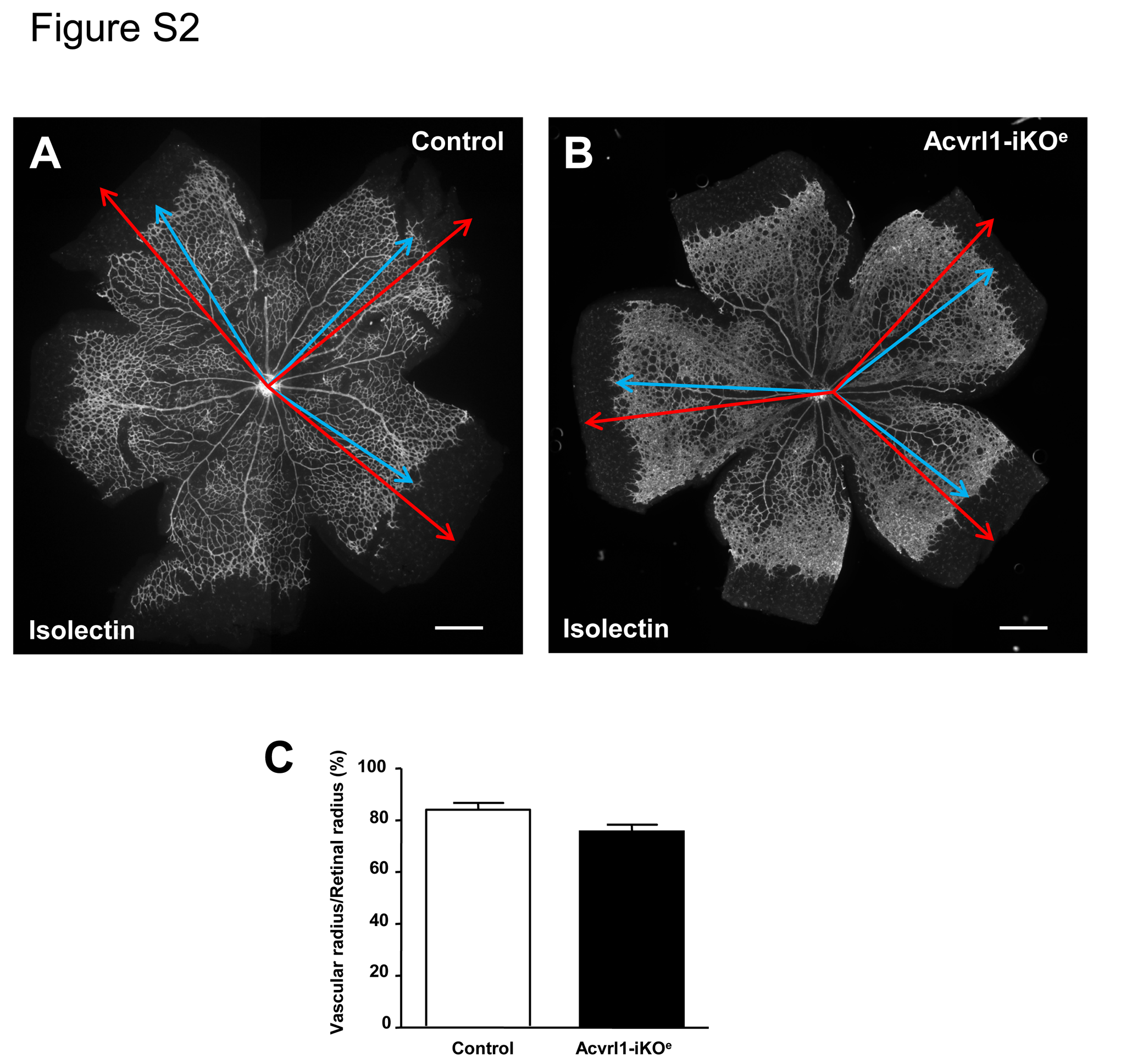

Supplement: Figure S2 — No reduction in progression of the retinal vascular plexus in neonatal Acvrl1-iKOe retinas. The relative distance covered by the vascular plexus was calculated as the ratio of the vascular radius, indicated by the blue arrow, and the retinal radius, indicated by the red arrow in 3 separate regions for each retina. The size of each retina was calculated as the mean of the retinal radii and mean values are shown +/− standard error. Scale bar = 500 um. (TIF) [file pone.0098646.s002.tif]

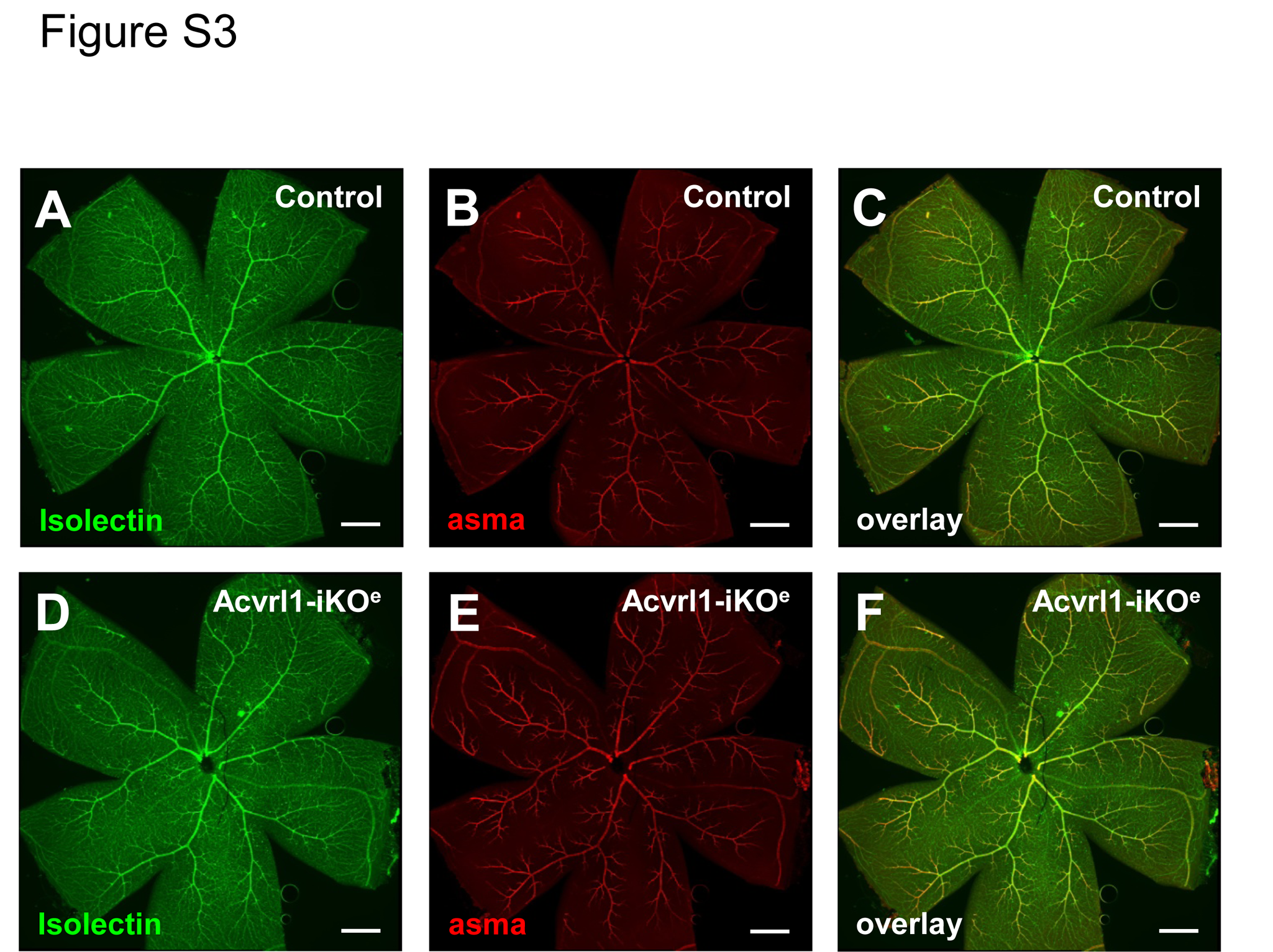

Supplement: Figure S3 — Normal vasculature of adult Acvrl1-iKOe retinas. No vascular abnormalities were detected in vessels from Acvrl1-iKOe (D–E) adult retinas compared with control retinas (A–C). Acvrl1 was depleted in endothelial cells during adult life and retinas were stained for vascular smooth muscle cells using anti-aSMA (red) and for ECs with isolectin (green). Scale bar = 500 um. (TIF) [file pone.0098646.s003.tif]

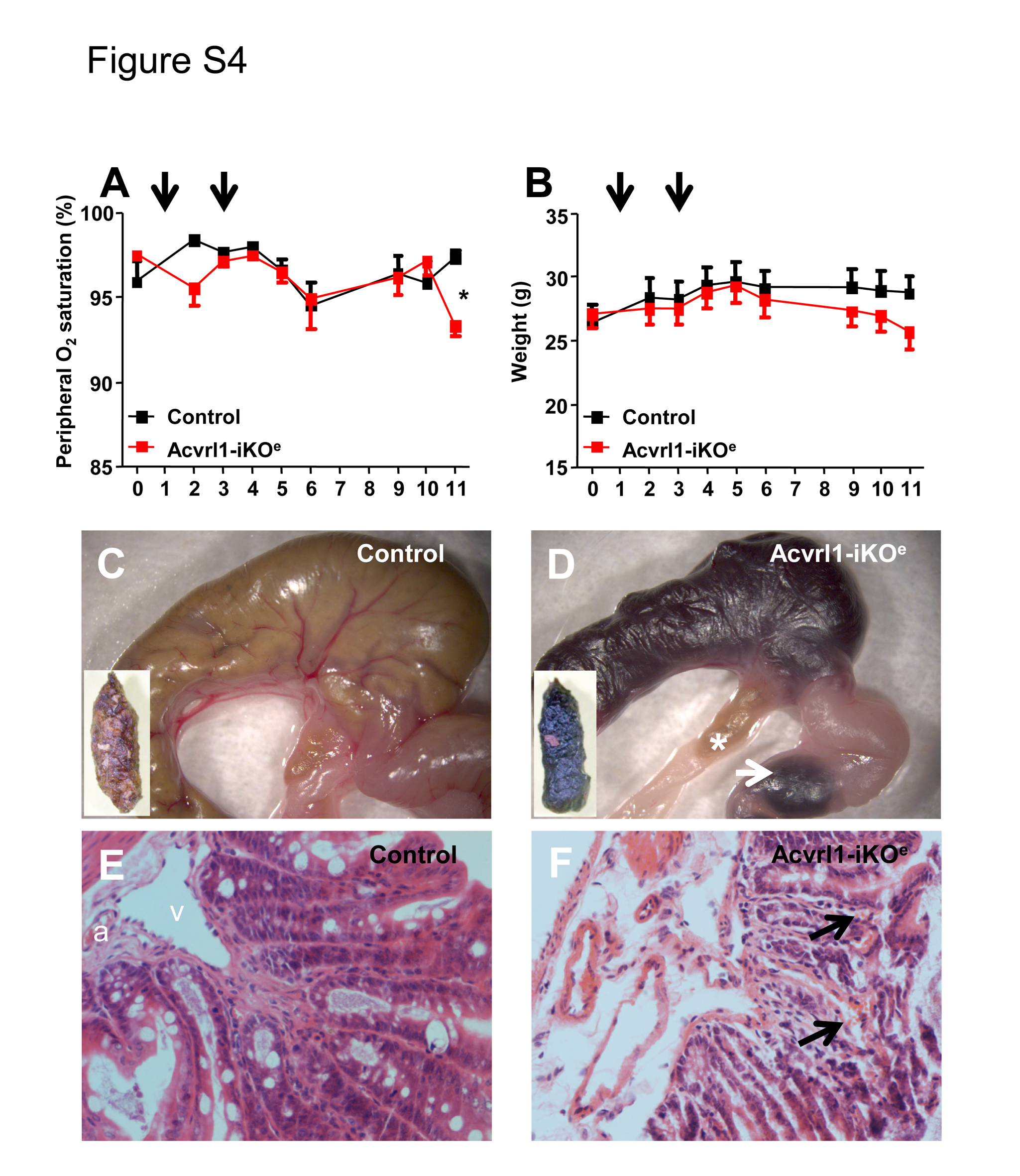

Supplement: Figure S4 — Adult Acvrl1-iKOe mice develop GI bleeding from the caecum. Tamoxifen was given to adult mice (on day 1 and day 3 as indicated by black arrows) to generate Acvrl1-iKOe mice. Peripheral oxygen saturation was significantly reduced in Acvrl1-iKOe adult mice at day 11* p<0.05 (A). GI bleeding was first observed by black faeces (inset in D), compare with normal faeces (inset in C), approximately 9 days after the first tamoxifen injection. Analysis of the GI tract showed bleeding was localised to the caecum, which appeared intensely dark coloured (D) compared with the caecum from control mice (C). Furthermore, the contents of the small intestine proximal to the caecum were normal in colour (asterisk in D), whilst the content of the large intestine immediately distal to the caecum was much darker indicating a significant blood content (arrow in D). H&E stained caecal sections revealed bleeding from fragile vessels in the caecal villi of adult Acvrl1-iKOe mice (arrows, F). (TIF) [file pone.0098646.s004.tif]
