# Supplementary material for: Endothelial Depletion of Acvrl1 in Mice Leads to Arteriovenous Malformations Associated with Reduced Endoglin Expression
Source: PLoS One. 2014 Jun 4;9(6):e98646. doi: 10.1371/journal.pone.0098646 (PMC4045906; doi:10.1371/journal.pone.0098646)
Supplement: Table S1 — Genes used in custom array qPCR analysis to evaluate changes in transcription following loss of Acvrl1. (* indicates housekeeping genes used for data normalisation). (DOCX) [file pone.0098646.s005.docx]

**Table S1 Genes used in custom array qPCR analysis.**

| **Gene** | **Full Name** | **Gene** | **Full Name** |
| --- | --- | --- | --- |
| **Acvrl1** | Activin A receptor, type II-like 1 | **Nfkb1** | Nuclear factor of kappa |
| **Acvr1b** | Activin A receptor, type 1B | **Nos3** | Nitric oxide synthase 3, endothelial cell |
| **Adam17** | A disintegrin and metallopeptidase domain 17 | **Notch4** | Notch gene homolog 4 |
| **Aldh1a2** | Aldehyde dehydrogenase family 1, subfamily A2 | **Notch1** | Notch gene homolog 1 |
| **Angpt1** | Angiopoietin 1 | **Nrp1** | Neuropilin 1 |
| **Angpt2** | Angiopoietin 2 | **Pdgfb** | Platelet derived growth factor, B polypeptide |
| **Angptl7** | Angiopoietin-like 7 | **Pgf** | Placental growth factor |
| **Arhgap21** | Rho GTPase activating protein 21 | **Ptgs2** | Prostaglandin-endoperoxide synthase 2 |
| **Atf2** | Activating transcription factor 2 | **Rbpj** | Recombination signal binding protein for  immunoglobulin kappa J region |
| **Cav1** | Caveolin 1, caveolae protein | **Sall1** | Sal-like 1 |
| **Ccl2** | Chemokine (C-C motif) ligand 2 | **Sall2** | Sal-like 2 |
| **Ccnd2** | Cyclin D2 | **Sall3** | Sal-like 3 |
| **Pecam1** | Platelet/endothelial cell adhesion molecule 1 | **Sall4** | Sal-like 4 |
| **Cdh2** | Cadherin 2 | **Sele** | Selectin, endothelial cell |
| **Cdh5** | Cadherin 5 | **Serpine1** | Serine (or cysteine) peptidase inhibitor, clade E, member 1 |
| **Cdkn1a** | Cyclin-dependent kinase inhibitor 1A (P21) | **Slc2a1** | Solute carrier family 2 member 1 |
| **Ctgf** | Connective tissue growth factor | **Smad6** | MAD homolog 6 (Drosophila) |
| **Cxcl2** | Chemokine (C-X-C motif) ligand 2 | **Smad7** | MAD homolog 7 (Drosophila) |
| **Cxcr4** | Chemokine (C-X-C motif) receptor 4 | **Snai1** | Snail homolog 1 (Drosophila) |
| **Dll4** | Delta-like 4 | **Snai2** | Snail homolog 2 (Drosophila) |
| **Edn1** | Endothelin 1 | **Sox17** | SRY-box containing gene 17 |
| **Eng** | Endoglin | **Srf** | Serum response factor |
| **Foxc1** | Forkhead box C1 | **Tgfbr1** | Transforming growth factor, beta receptor I |
| **Foxc2** | Forkhead box C2 | **Tgfbr2** | Transforming growth factor, beta receptor II |
| **Foxh1** | Forkhead box H1 | **Tgfbr3** | Transforming growth factor, beta receptor III |
| **Fzd4** | Frizzled homolog 4 (Drosophila) | **Thbs1** | Thrombospondin 1 |
| **Frzb** | Frizzled-related protein | **Thbs2** | Thrombospondin 2 |
| **Gpr124** | G protein-coupled receptor 124 | **Twist1** | Twist homolog 1 |
| **Hes1** | Hairy and enhancer of split 1 | **Vegfa** | Vascular endothelial growth factor A |
| **Hey1** | Hairy/enhancer-of-split related with YRPW motif 1 | **Vegfb** | Vascular endothelial growth factor B |
| **Htra1** | HtrA serine peptidase 1 | **Vegfc** | Vascular endothelial growth factor C |
| **Id1** | Inhibitor of DNA binding 1 | **Flt1** | FMS-like tyrosine kinase 1 |
| **Id2** | Inhibitor of DNA binding 2 | **Kdr** | Kinase insert domain protein receptor |
| **Id3** | Inhibitor of DNA binding 3 | **Flt4** | FMS-like tyrosine kinase 4 |
| **Itgav** | Integrin alpha V | **Wnt10b** | Wingless related MMTV integration site 10b |
| **Itgb8** | Integrin beta 8 | **Wnt5a** | Wingless-related MMTV integration site 5A |
| **Klf6** | Kruppel-like factor 6 | **Wnt7a** | Wingless-related MMTV integration site 7A |
| **Lrp5** | Low density lipoprotein receptor-related protein 5 | **Wnt7b** | Wingless-related MMTV integration site 7B |
| **Mgp** | Matrix Gla protein | **Krit1** | KRIT1, ankyrin repeat containing |
| **Mmp14** | Matrix metallopeptidase 14 (membrane-inserted) | **S100a6** | S100 calcium binding protein A6 (calcyclin) |
| **Mmp2** | Matrix metallopeptidase 2 | **Icam2** | Intercellular adhesion molecule 2 |
| **Mmp9** | Matrix metallopeptidase 9 | **B2m*** | Beta-2 microglobulin |
| **Mpz** | Myelin protein zero | **Gapdh*** | Glyceraldehyde-3-phosphate dehydrogenase |
| **Myc** | Myelocytomatosis oncogene | **Actb*** | Actin, beta |

(* housekeeping genes used for data normalisation)
