# Supplementary material for: Endothelial Depletion of Acvrl1 in Mice Leads to Arteriovenous Malformations Associated with Reduced Endoglin Expression
Source: PLoS One. 2014 Jun 4;9(6):e98646. doi: 10.1371/journal.pone.0098646 (PMC4045906; doi:10.1371/journal.pone.0098646)
Supplement: Table S2 — Blood Gas Analysis of Acvrl1-iKOe Adult Mice. Blood from adult male Acvrl1-iKOe and control mice (aged 12 weeks) was taken by cardiac puncture under terminal anaesthesia and analysed using a CG8+ cartridge with an Istat portable reader. Blood values are expressed as mean ±SEM. (DOCX) [file pone.0098646.s006.docx]

**Table S2**

**Blood Gas Analysis of Acvrl1-iKO^e^ Adult Mice**

|  | Control | Acvrl1-iKO^e^ | p value |
| --- | --- | --- | --- |
| n | 6 | 5 |  |
| pH | 7.29±0.01 | 7.22±0.01 | 0.03 |
| PCO2, mmHg | 46.2±2.9 | 51.2±1.9 | 0.54 |
| PO2, mmHg | 61.8±1.7 | 61±7.7 | 0.93 |
| Base Excess | -4.8±1 | -6.8±0.7 | 0.19 |
| HCO3, nmol/L | 21.9±1. | 20.9±0.5 | 0.58 |
| TCO2, nmol/L | 23.5±1.8 | 22.2±0.6 | 0.40 |
| Mean SaO_2_, % | 87.8±1.8 | 82.6±6.6 | 1 |
| Na, nmol/L | 143.7±0.6 | 140.2±2.2 | 0.12 |
| K, nmol/L | 5.2±0.3 | 6 ±0.6 | 0.31 |
| iCa, nmol/L | 1.2±0.03 | 1.2±0.03 | 0.93 |
| Glucose, mg/dL | 237±19 | 194±25 | 0.25 |
| Hct, % PCV | 35.6±1 | 13.2±2.5 | 0.008 |
| Hb, g/dL | 12.1±0.3 | 4.5±0.9 | 0.008 |
